# Vaccines against COVID-19: Priority to mRNA-Based Formulations

**DOI:** 10.3390/cells10102716

**Published:** 2021-10-11

**Authors:** Steve Pascolo

**Affiliations:** Department of Dermatology, University Hospital of Zürich, Raemistrasse 100, 8091 Zürich, Switzerland; steve.pascolo@usz.ch

**Keywords:** mRNA, protein, adenovirus, SARS-CoV-2, vaccine, spike

## Abstract

As of September 2021, twenty-one anti-COVID-19 vaccines have been approved in the world. Their utilization will expedite an end to the current pandemic. Besides the usual vaccine formats that include inactivated viruses (eight approved vaccines) and protein-based vaccines (four approved vaccines), three new formats have been validated: recombinant adenovirus (six approved vaccines), DNA (one approved vaccine), and messenger RNA (mRNA, two approved vaccines). The latter was the fastest (authorized in 2020 in the EU, the USA, and Switzerland). Most Western countries have reserved or use the protein vaccines, the adenovirus vaccines, and mRNA vaccines. I describe here the different vaccine formats in the context of COVID-19, detail the three formats that are chiefly reserved or used in Europe, Canada, and the USA, and discuss why the mRNA vaccines appear to be the superior format.

## 1. Introduction

Since the validation by Jenner of the principle of vaccination (using cow- (“vacca”) pox virus as a prophylaxis for smallpox), attenuated and inactivated viruses have been the components of efficacious vaccines. Their production, which requires usually animal cells (or eggs), can be difficult (particularly if the virus is lytic), and their purification cannot be too stringent as it would destroy their integrity. As a consequence, the vaccines based on whole viruses contain impurities from the production process that may cause problems, in terms of the induction of irrelevant immunity and intolerance/allergies. Subunit vaccines have proven to be safe and efficacious and somewhat easier to produce, purify, and store. They, however, must be complemented with adjuvants. More recently, recombinant attenuated viruses have been generated that rely on robust production, safety, and efficacy features established for modified attenuated viruses (for example, adenoviruses). This type of vaccine was first approved in summer 2020 for protection against Ebola https://johnsonandjohnson.gcs-web.com/static-files/1c979f4f-cad3-4f8b-9a22-69aaac503570 (accessed on 7 October 2021). Finally, since the end of the 1990s, companies (starting with the one I cofounded: CureVac) are developing vaccines based on synthetic, in vitro-transcribed mRNA (ivt mRNA) [1,2,3,4,5]. This format, first published by a French team in 1993 [6], confirmed later by an American team [7] and a German team [8], was originally neglected because mRNA was believed to be a fragile molecule. This is a misconception. Because of the omnipresence of RNases, research and development with mRNA must be conducted in special “RNase-free” conditions. However, the mRNA molecules themselves are physiochemically very stable in the absence of RNases and at neutral to acidic pH (to avoid deprotonation of the 2′OH, which could result in ester cleavage of the RNA backbone). They can be frozen, thawed, lyophilized, and resuspended without losing any functionality [1]. As a matter of fact, mRNA is the only biological macromolecule that can be heated up to 95 °C without losing its activity [5]. Other biological macromolecules such as DNA, proteins, or also viruses lose their functionality if they are heated up to the same high temperatures (even if, in controlled laboratory conditions, proper refolding of proteins or annealing of DNA strands could take place after denaturation using precisely established cooling methods). Thus, counterintuitively, mRNA is the most stable biological molecule for the production of vaccines (the liposomes used to generate mRNA vaccines may not be stable; that is why the current mRNA vaccines have to be stored at low temperatures). The European Union has recognized this feature by awarding CureVac, in 2014, a two-million Euro prize for vaccines stable at room temperature (https://ec.europa.eu/commission/presscorner/detail/en/IP_14_229 accessed on 7 October 2021). Still, the prejudice that mRNA would be instable remained strongly present in the psyche of the scientific and medical communities, which plagued the development of mRNA-based drugs for the last thirty years. The COVID-19 pandemic created a definitive U-turn in this bias and allowed the mRNA vaccine to showcase its potential (i.e., its speed/ease of production, safety, and efficacy), which made it the first vaccine format approved against SARS-CoV-2 infections, less than one year after the publication of the viral sequence [9]. After this tour-de-force, some recombinant attenuated adenovirus, inactivated SARS-CoV-2, and protein-based anti-SARS-CoV-2 vaccines have also been approved, and this gives the world currently a panel of 21 prophylactic weapons against the pandemic (https://covid19.trackvaccines.org/vaccines/ accessed on 7 October 2021).

## 2. Are All Anti-COVID-19 Vaccines Similar in Their Safety and Efficacy Profile?

Concerning safety, as they all passed the evaluation processes, they all have been approved as safe (even if, as expected for vaccines, side effects have been recorded, such as frequent fatigue and local reactogenicity; rare allergies could be observed). However, are there also theoretical concerns that should be mentioned?

Concerning efficacy, all approved vaccines protect well against COVID-19 (more than 70%, which is higher than most flu vaccines, and some up to over 95%). Although all current recombinant vaccines use the SARS-CoV-2 spike protein as a target, some use the wild-type protein (for example, AstraZeneca’s adenovirus vaccine), while others use a mutated version of it (for example, the vaccines from Moderna and BioNTech/Pfizer), in particular one that contains two consecutive prolines (lysine 986 and valine 987 are both replaced with prolines: “PP Spike”). This was previously shown for the SARS-CoV and MERS-CoV spikes to keep the protein in a prefusion conformation (the conformation at the virus surface) [10].

## 3. Anti-COVID-19 Vaccines

Five vaccine platforms are currently providing approved vaccines against SARS-CoV-2:

1. Vaccines based on ivt mRNA developed primarily in Europe and the U.S. by BioNTech/Pfizer and Moderna;
2. Vaccines based on recombinant deficient adenoviruses optimized and produced in different countries and by companies including AstraZeneca, Johnson & Johnson/Janssen, and CanSino;
3. Protein-based vaccines mostly advanced in the U.S. and Russia;
4. Inactivated SARS-CoV-2 viruses developed mostly in China and India;
5. A DNA vaccine only approved in India.

In addition, promising new experimental vaccination approaches such as, for example, aerosolized adenovirus [11], adeno-associated viral (AAV)-based vaccines [12], live attenuated vaccines that can be applied intranasally [13,14], or other vector vaccines such as modified vaccinia virus Ankara (MVA)-based approaches [15] are being developed. However, they will not be further detailed here. Meanwhile, little information is available about inactivated SARS-CoV-2 and DNA. Thus, I will present in the following the features of the three platforms (ivt mRNA, recombinant adenovirus, and purified protein) for which most Western countries have reserved millions of doses. Table 1 presents the advantages and disadvantages (in some cases, only theoretical) of the four platforms, and Table 2 presents the results obtained against COVID-19.

## 4. Protein-Based Vaccines

A safety advantage of proteins is that they are inert and naturally eliminated. However, when produced in cell culture, they contain contaminants coming from the cultures [16]. This must be kept in mind particularly when allergic patients are vaccinated. In addition, protein-based vaccines require an adjuvant. The design and production of a protein for a vaccine can be somewhat cumbersome as every protein is different (by its structure and glycosylation status and whether it is hydrophilic or hydrophobic). Thus, obtaining and preserving the correct antigenic features of the purified protein in the vaccine can be a challenge. Should the vaccine contain immunogenic impurities (including misfolded antigens), it could induce an irrelevant immune response. The protein vaccine from Novavax contains a recombinant full-length SARS-CoV-2 PP spike (also mutated in positions 682 to 685 in order to confer protease resistance) that is produced in insect cells and mixed with Matrix-M1, a saponin-based adjuvant. It is stored at 2 °C to 8 °C. The tested doses were 5 μg and 25 μg of protein per injection and were found to induce similar high titers of neutralizing antibodies in phase I trials [17]. The phase III trial was performed with two injections of 5 μg SARS-CoV-2 rS + 50 μg Matrix-M1 adjuvant 21 d apart. The company reported in January 2021 an 89.3% efficacy against COVID-19 (95.6% against the original COVID-19 strain and 85.6% against the alpha variant strain B.1.1.7 identified in the U.K.). Those data are confirmed in a recent publication: efficacy of 86.3% against the alpha variant and 96.4% against non-alpha variants [18]. Thus, the Novavax vaccine appears as an efficient and safe product of great interest. Another protein vaccine (EpiVacCorona) is available and approved in Russia. However, little information is available (https://clinicaltrials.gov/ct2/show/NCT04527575 accessed on 7 October2021). Nevertheless, it could be of interest as it is not produced by cells, but chemically synthesized. The chemical peptide antigens corresponding to the SARS-CoV-2 protein are conjugated to a carrier protein and adsorbed on an aluminum hydroxide. These types of chemically synthesized vaccines would not contain contaminants such as proteins from the producing cells in vitro and therefore present less risk of inducing allergies or irrelevant immunity.

## 5. Adenovirus-Based Vaccines

Adenoviruses are nonenveloped viruses that are particularly resistant to chemical or physical agents. Replication-defective adenoviruses are used to generate recombinant vaccines [19]. The recombinant DNA contained in these viruses is actively sent to the nucleus of mammalian cells, where it can be transcribed into mRNA. Usually, vaccine adenoviruses are deleted of the early gene E1 so that they can infect cells, but not replicate. Their production is possible thanks to complementation in cells that express E1. The AstraZeneca vaccine is produced in HEK293 cell lines that are derived from human embryonic kidney cells (taken from an aborted female fetus in 1973) transformed by integration of a part of adenovirus 5 that also gives the expression of the E1 proteins. Thanks to this protein, HEK293 can produce viruses that are deficient for E1. The gene coding for the antigen (here, the SARS-CoV-2 spike) is introduced into these replication-deficient viruses, rendering them into recombinant replication-defective (thereby attenuated) vaccine viruses. The production of adenoviruses requires cell culture and optimization of this process. Theoretical concerns include whether the recombinant defective adenovirus could recombine/evolve in vitro during production (not all viruses collected from cell culture would have the same sequences) or in vivo after injection (for example, in a person being infected with a common adenovirus), leading to the production of new adenoviruses. Another concern is the persistence and integration of the chimeric DNA in the human genome. DNA viruses are known to eventually integrate into one out of one million cells in vivo in mice for adenoviruses [20]. In addition, it is not known how the DNA sequence of the SARS-CoV-2 spike (which normally does not exist in nature since coronaviruses are RNA viruses) would behave in cells and whether it could influence persistence and integration rates (one could speculate by sheer unfortunate chance that the SARS-CoV-2 spike DNA sequence could be stabilized by peculiar DNA-specific proteins such as special histones or by DNA recombination machineries; however, so far, no evidence supporting this theory has been reported). The recombinant adenoviruses combine several elements from several viruses that would not have met in nature, and the long-term outcome in vivo of such chimeras is still to be determined.

Among the approved adenovirus-based vaccines, the one primarily utilized and most widely distributed so far is from AstraZeneca and named ChAdOx1 nCoV-19 (AZD1222). It is a recombinant replication-deficient chimpanzee adenovirus in which a promoter from cytomegalovirus was introduced before a gene sequence from the tissue plasminogen activator (the leader sequence); it also contains the cDNA coding for the full-length wild-type spike and, at the end, a bovine growth hormone polyadenylation sequence. The vaccine (5 × 10^10^ particles per injection, which means approximately 1.5 µg of DNA) is administered intramuscularly in two doses, given between 4 wk and 12 wk apart. It was reported to give a different level of protection (depending on the dose and the clinical trial center), but the overall efficacy was above 70% [21]. Although it protects against the alpha variant, it does not seem to protect well against the beta variant (B.1.351 identified in South Africa) [22]. Several countries (including Denmark, Austria, Estonia, Latvia, and Luxemburg) have suspended vaccination with ChAdOx1 nCoV-19 because of cases of thrombosis seen after vaccination. The link between the vaccine and these symptoms has been established [23]. Another attenuated recombinant adenovirus that is broadly approved as a vaccine against SARS-CoV-2 is Sputnik V (Gamaleya Research Institute), developed in Russia. It consists of two replication-deficient adenoviruses (recombinant adenovirus type 26 and recombinant adenovirus type 5, given sequentially), both of which carry the gene for the full-length wild-type spike (rAd26-S and rAd5-S). The vaccine is administered at a dose of 10 11 particles intramuscularly in two doses 21 d apart and provides 91.6% efficacy [24]. Meanwhile, the approved Johnson & Johnson/Janssen recombinant adenovirus vaccine Ad26.COV2.S was selected among seven experimental recombinant adenovirus serotype 26 (Ad26) vector-based vaccines. It notably also differs from the AstraZeneca vaccine because it encodes for a spike that has the two prolines stabilizing the prefusion conformation. A single intramuscular shot of 5 × 1010 particles provided 66% protection against moderate and severe COVID-19 even in South Africa, where the (beta) B.1.351 variant is prevalent [25] (https://www.fda.gov/media/146265/download accessed on 7 October 2021).

## 6. Nonreplicating ivt mRNA Vaccines

**Figure 1:**
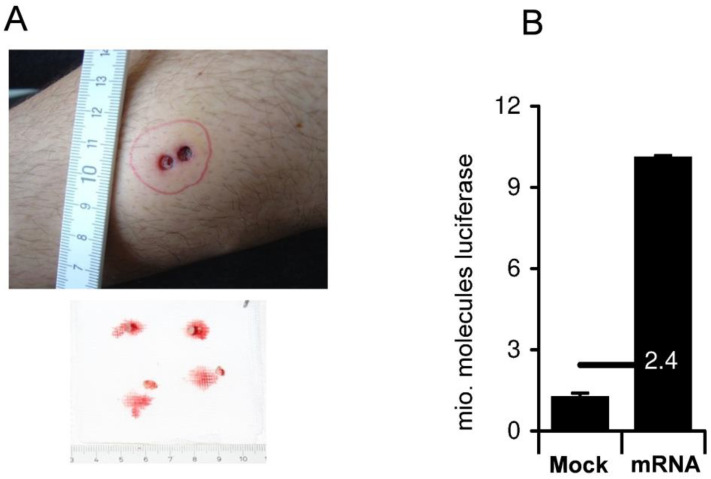
The first injection of ivt mRNA coding luciferase in human skin as published in 2007 [29]. In **A**, upper panel, the area of my skin, which was the site of intradermal administration of 120 μg of unmodified synthetic mRNA coding for luciferase, is circled in red (evident by the bubble formed by the intradermal injection). Seventeen hours later, under local anesthesia, three-millimeter punch biopsies (lower panel) were taken in (as seen in the upper panel) and outside (not shown) the mRNA injection site, as well as in PBS (“Mock”) injected sites. The skin is not inflamed, and there were no systemic side effects, showing that injection of unmodified synthetic mRNA is safe in humans. The measurement of luciferase activity in these biopsies (**A**, lower panel), as well as in other biopsies taken from other sites of my skin distant from the site of mRNA injection (“Mock”) demonstrated that naked synthetic mRNA could be taken up and expressed in human skin (in **B**; data similar to what we published previously [5]). The experiment was repeated three times between 2003 and 2005. Only biopsies within the area receiving mRNA (delimited in red) were emitting luminescence above background levels in the presence of luciferin substrate. These results demonstrated that the data generated in mice (the surprising uptake and expression of injected naked ivt mRNA) is not restricted to this organism, but can be extended to humans. As written in the article reporting these results in 2007, “A qualified healthy individual volunteered for intradermal injections of 150 μL of a Ringer lactate solution containing the GMP quality RNActive coding for luciferase. Before performing the experiment, a letter of consent was signed by the volunteer.”. The injections, anesthesias, and punch biopsies were performed by a medical doctor in the Dermatology Department at the University Clinic of Tuebingen and according to German regulations.

The main feature of nonreplicating ivt mRNA vaccines is their safety. Indeed, this natural molecule cannot replicate, is active in the cytosol, and is quickly and fully degraded by abundant RNases. Thus, as opposed to recombinant adenoviruses, but similar to proteins, this vaccine format has no risk to persist, recombine, or modify the host genome [1,2,3,4]. As we have shown in our 2006-certified first worldwide pharmaceutical production plant in Tuebingen, Germany, synthetic mRNA can be easily produced at large scales and highly purified by HPLC [5]. Indeed, following on from the project imagined by Prof. Rammensee in Tuebingen in 1996 and executed mostly by Dr. Ingmar Hoerr (as part of his Ph.D. training) [8], I started testing and optimizing synthetic mRNA vaccines in 1998 (at that time, I was a postdoctoral researcher in Prof. Rammensee’s laboratory) in wild-type and humanized mice (HHD) that I developed during my Ph.D. at the Pasteur Institute in France [26,27]. Initial experiments indicated that this format works as an anticancer vaccine and antiviral vaccine; however, immune responses were usually weaker than those induced by other vaccine formats, for example plasmid DNA. The safety advantage of mRNA (being quickly degraded) stimulated us to further optimize this format with the goal of bringing it to the clinic. To this end, we studied the parameters that are important for optimizing the mRNA vaccine (i.e., stability of the mRNA [28], expression of the encoded protein [29], HPLC purification [1], formulation [30], and immunostimulating capacities of RNA [31,32]) and, as a result, implemented the first worldwide pharmaceutical manufacturing of synthetic in vitro transcribed (ivt) mRNA [1]. Starting in 2003, we also conducted, with the Dermatology and Haemato-oncology Departments of Tuebingen’s University Hospital, the first studies in man, evaluating the expression of synthetic mRNA injected in my own skin (Figure 1) [29], and the safety and efficacy of anticancer vaccines based on synthetic mRNA [33,34,35].

Improvements of the ivt mRNA production, design, and structure these last 20 y have transformed this molecule into a very efficient genetic vehicle for vaccines and therapies [36,37]. Of note, a revolutionary Cap analog named CleanCap has largely improved the production and efficacy of synthetic mRNA [38]. The ivt mRNA encodes a single antigen, limiting the risk of triggering immunity against irrelevant antigens, as can be seen with proteins (i.e., contaminants, misfolded proteins) or adenoviruses (vector proteins). The synthetic mRNA used in the anti-COVID-19 vaccines is condensed in liposomes consisting usually of four different lipids and related to the other RNA (not an mRNA, but a small inhibitory RNA, siRNA) liposomal formulation approved in 2018: ONPATTRO (injected intravenous at 30 mg per dose intravenous every 3 wk for the treatment of polyneuropathy, caused by hereditary transthyretin-mediated amyloidosis). One of the qualities of the ivt mRNA vaccine is the speed and ease of production: any DNA sequence preceded by an adequate promoter (usually from the T7 or SP6 bacteriophages) is efficiently transcribed in vitro using the recombinant RNA polymerase (usually T7 or SP6 [39]) [5]. One molecule of DNA can give over one-thousand molecules of mRNA within a few hours. The production of mRNA required for one million doses of an ivt mRNA vaccine can be achieved in 6 L of in vitro transcription, while the production of viruses for one million vaccine doses typically requires 6000 L of cell culture [5]. All products in the transcription reaction (DNA, RNA polymerase, nucleotides, Cap analog) are from bacteria or chemical origins. Thus, the ivt mRNA vaccine is “vegan”. Besides overcoming religious or behavior limits, this feature also reduces the risk of allergies or the development of irrelevant immunity against contaminants from cell cultures. As we discovered in early 2000, RNA is a danger signal that triggers Toll-like receptors [31,32,40]. Thus, (unmodified) mRNA-based vaccines contain an intrinsic adjuvant. The induction of inflammation is required to trigger the development of an immune response after injection of a formulation.

Although the utilization of synthetic mRNA in vaccines is relatively new (described first in 1993 [6], injected in patients [33,34,35] and myself (Figure 1) [29] in the mid-2000s, and approved first in 2020 [9]), the utilization of natural mRNA to vaccinate is ancient: the yellow fever, mumps, measles, and rubella vaccines are attenuated RNA viruses that immunize after subcutaneous injection, by delivering their mRNA into the host cells, which subsequently produce viral particles that initiate an immune response. These ancient vaccines can therefore be considered “natural” mRNA vaccines, while the newly approved anti-SARS-CoV-2 vaccines are “synthetic” mRNA vaccines. However, they both rely on the same fundamental mechanisms: the production of viral proteins by host cells using injected mRNA. The optimization of the ivt mRNA molecules and of the liposomal formulations have turned the ivt mRNA vaccine into a very potent format [37,41]. It has been largely evaluated in clinical trials mostly as a vaccine against cancer [42,43]. However, it has not yet demonstrated efficacy in cancer treatment as a pivotal study in prostate carcinoma by CureVac did not demonstrate a survival advantage in vaccinated patients. Meanwhile, BioNTech together with Genentech is currently performing a phase II study with an individualized mRNA vaccine coding for mutations identified in tumor biopsies. Each patient receives his/her own tailored vaccine (http://merit-consortium.eu/ accessed on 7 October 2021). Thus, it can be expected that anticancer mRNA vaccines, particularly when used in combination with other treatments (chemotherapy, radiotherapy, immunotherapy), will soon be approved.

In the context of COVID-19, in the first half of 2020, five nonreplicating mRNA vaccines were produced and evaluated: three by BioNTech (BNT162a1, which is made with unmodified nucleotides; BNT162b1 [44,45,46] and BNT162b2 [9], which contain pseudo-uridine, the “1” series coding for the receptor binding domain of spike, while the “2” codes for the full-length spike), one by CureVac (CvNCoV, which is made with unmodified nucleotides and codes the full-length spike [47] (preprint at https://papers.ssrn.com/sol3/papers.cfm?abstract_id=3911826 accessed on 7 October 2021), and one by Moderna (which contains pseudo-uridine and codes the full-length spike [48]). The results obtained in humans with the unmodified mRNA vaccine BNT162a1 are not yet published. Originally, the pseudo-uridine modification was coined in 2005 to abrogate innate immunostimulation by RNA (triggering of Toll-like receptors) [49], allowing the use of ivt mRNA for nonimmunogenic (nonvaccinal) expression of protein. However, it was published in 2017 that, surprisingly, even modified mRNA can be used in mRNA vaccines [50]. The adjuvant effects in this case probably come from the lipids used in the liposomal formulations (the liposomes are similar in BioNTech/Pfizer, CureVac, and Moderna, although small variations in some of the lipids characterize each of them as presented in the review [36]). Whether modified (pseudo-uridine) or unmodified mRNA is better for an ivt mRNA vaccine is not yet established and may largely depend on formulation, dose, site of injection and intended aim (e.g., induction of antibodies and/or T-cells; targeting infectious agent, allergy or cancer). Injections of unmodified RNA induces production of type I interferon (stimulation of, e.g., TLR7) that may induce the blockade of the translation and degradation of mRNA. On this basis, it is proposed that modified mRNA would trigger a better adaptive immune response thanks to a higher translation and stability of the foreign mRNA. However, two parameters would argue against this hypothesis: (i) not all cells respond to type I interferon, and the immune system has developed specialized antigen-presenting cells (characterized by the expression of USP18) that maintain protein translation even in the presence of type I interferons, in order to further stimulate lymphocytes in the context of an infection [51]; (ii) type I interferon was reported to be important for effective and long-lived adaptive immunity (review in [37]). Thus, it remains to be determined whether for the induction of the most efficient and long-lived anti-COVID-19 immunity, an unmodified or a modified mRNA should be used in the vaccine formulations.

Although the first injections of an anti-SARS-CoV-2 ivt mRNA vaccine (and the first worldwide injection in humans of an experimental anti-SARS-CoV-2 vaccine) in volunteers was conducted by Moderna on March 16 2020, the first approval was for the mRNA vaccine of BioNTech in association with Pfizer (Comirnaty^®^) in December 2020. These two mRNA vaccines give over 90% protection against COVID-19 [9,48] and protect against emerging variants (although the neutralization of the beta variant requires lower dilutions of sera than those required to neutralize the initial virus and other variants) [52]. CureVac’s unmodified mRNA vaccine gives an efficacy of 48.2% against symptomatic COVID-19 but interestingly an efficacy of 70.7% against moderate-to-severe COVID-19 (preprint at https://papers.ssrn.com/sol3/papers.cfm?abstract_id=3911826 accessed on 7 October 2021). In the age group of 18 to 60, protection against moderate to severe disease was calculated to be 77%. The company seeks regulatory approval in the segment of the population where it provides protection (https://www.curevac.com/en/2021/08/31/curevacs-cvncov-phase-2b-3-study-data-published-in-preprints-with-the-lancet/ accessed on 7 October 2021). In Israel, where a majority of the population has been vaccinated using the BioNTech/Pfizer or Moderna vaccine, a study on over 10,000,000 persons (596,618 vaccinated and 596,618 nonvaccinated) demonstrated a vaccine efficacy of 92% against infection, 94% against symptomatic COVID-19, 87% against hospitalization, and 92% against severe disease (determined 7 d or more after the second dose). Thus, the approved mRNA vaccines are highly efficacious, not only in preventing the disease, but also transmission [53].

Thanks to the upgraded production capacities of BioNTech and Moderna (the implementation of new and large mRNA production factories: in Visp, Switzerland, for Moderna—in collaboration with Lonza—and Marburg, Germany for BioNTech.), it is expected that there will be enough synthetic mRNA vaccine doses to fully, safely, and efficiently vaccinate a large part of the Western world’s population before the end of 2021.

## 7. Conclusions

Although, as expected for vaccines that strongly activate the immune system, the approved vaccines against SARS-CoV-2 have frequent side effects (mostly fatigue, headaches, and local reactogenicity), they all provide efficient protection against COVID-19 and are widely administered in order to end the pandemic. Three vaccine formats are largely approved in the world. The ivt mRNA vaccine combines the safety aspects of the traditional protein-based vaccines (inert and quickly eliminated), while it has the versatility of recombinant viruses. However, as opposed to recombinant adenoviruses, ivt mRNA-based vaccines do not have the risk of evolving, recombining, or integrating in genomes, and as opposed to proteins, the risk of inducing immunity against contaminants is limited. The safety and flexibility aspects of the ivt mRNA vaccines have turned them into the winners of the race to develop vaccines against COVID-19. These features also keep this format optimal in terms of the design and production of vaccines that might be needed in case variants of SARS-CoV-2 resisting the current vaccine-induced immune response emerge (this is so far not the case even if the beta variant is less well recognized than other variants by mRNA-vaccine-induced antibodies). As the vaccines induced immune response declines over time [54], a third dose more than six months after the second dose is recommended, particularly in elderly people or at-risk groups. In Israel, although a fourth wave of COVID came in summer 2021, it is estimated that, altogether, two thirds of hospitalizations and deaths have been prevented by the mRNA vaccine campaign [55]. This illustrates the great benefits brought by the anti-COVID mRNA vaccines. Thanks to their safety and ease of production, mRNA vaccines, as well as mRNA-based therapies are being intensively developed with the promise to create new drugs against many different diseases.

## Figures and Tables

**Table 1:** Advantages and disadvantages of selected vaccine platforms.

	Design	Upscaling	Re-Using Established GMP Conditions	Theoretical Safety Concerns
Recombinant viral vector (adenovirus)	Not easy	To be optimized	Not guaranteed	Recombination/persistence/integration
ivt mRNA	Easy	Easy	Guaranteed	None
Proteins/ sugars	Not easy	To be optimized	Not guaranteed	None
Inactivated viruses	Easy	To be optimized	Not guaranteed	None

**Table 2:** Vaccines against COVID-19 chiefly ordered by Westernized countries. * Either the wild-type sequence of the SARS-CoV-2 spike (WT) or the modified sequence containing two consecutive prolines (PP) is used.

Platform	Company	Spike *	Efficacy (January 2020 SARS-CoV-2)	Efficacy (Beta Variant ^&^)	Dosing	Theoretical Concerns
Purified protein	Novavax #	PP	95%	Reduced	5 μg 2× with 3-week interval	Induction of immunity against contaminants
	Sanofi-GSK #	PP	Not yet known	Not yet known	Undisclosed	
Recombinant adenovirus	AstraZeneca	WT	Between 62% and 90%	Strongly reduced	ca. 2 μg 2× with 4-week interval	Recombination Integration in genome
	J&J/Janssen	PP	72%	Not reduced	ca. 2 μg once	
ivt mRNA	BioNTech /Pfizer	PP	95%	Slightly reduced	30 μg 2× with 3-week interval	None
	CureVac #	PP	47% *	Not yet known	12 μg 2× with 4-week interval	
	Moderna	PP	94.1%	Slightly reduced	100 μg 2× with 4-week interval	

* The CureVac vaccine was tested later than the others and, as a consequence, in the context of numerous variants. # Vaccine not yet approved. ^&^ Data for all variants of concern are not yet available for each vaccine; thus, the description is limited here to the beta variant.
